# Epimedium flavonoids improve cerebral white matter lesions by inhibiting neuroinflammation and activating neurotrophic factor signal pathways in spontaneously hypertensive rats

**DOI:** 10.1002/alz.087158

**Published:** 2025-01-09

**Authors:** Lan Zhang, Lin Li, Denglei Ma, Weipeng Wei

**Affiliations:** ^1^ Xuanwu Hospital of Capital Medical University, Beijing, Beijing China; ^2^ Xuanwu Hospital of Capital Medical University, Beijing China

## Abstract

**Background:**

Cerebral small vessel disease (CSVD) is one of the most common nervous system diseases. Hypertension and neuroinflammation are considered important risk factors for the development of CSVD and white matter (WM) lesions.

**Method:**

We used the spontaneously hypertensive rat (SHR) as a model of early‐onset CSVD and administered epimedium flavonoids (EF) for three months. The learning and memorization abilities were tested by new object recognition test. The pathological changes of WM were assessed using magnetic resonance imaging, transmission electron microscopy (TEM), Luxol fast blue and Black Gold staining. Oligodendrocytes (OLs) and myelin basic protein were detected by immunohistochemistry. The ultrastructure of the tight junctions was examined using TEM. Microglia and astrocytes were detected by immunofluorescence. RNA‐seq was performed on the corpus callosum of rats.

**Result:**

The results revealed that EF could significantly improve the learning and memory impairments in SHR, alleviate the injury and demyelination of WM nerve fibers, promote the differentiation of oligodendrocyte precursor cells (OPCs) into mature OLs, inhibit the activation of microglia and astrocytes, inhibit the expression of p38 MAPK/NF‐κB p65/NLRP3 and inflammatory cytokines, and increase the expression of tight‐junction related proteins ZO‐1, occludin, and claudin‐5. RNA‐seq analysis showed that the neurotrophin signaling pathway played an important role in the disease. RT‐qPCR and WB results showed that EF could regulate the expression of nerve growth factor and brain‐derived neurotrophic factor and their downstream related proteins in the neurotrophin signaling pathway.

**Conclusion:**

This study might explain the potential mechanism of EF’s effects on the cognitive impairment and WM damage caused by hypertension.

